# HiChIP: a high-throughput pipeline for integrative analysis of ChIP-Seq data

**DOI:** 10.1186/1471-2105-15-280

**Published:** 2014-08-15

**Authors:** Huihuang Yan, Jared Evans, Mike Kalmbach, Raymond Moore, Sumit Middha, Stanislav Luban, Liguo Wang, Aditya Bhagwate, Ying Li, Zhifu Sun, Xianfeng Chen, Jean-Pierre A Kocher

**Affiliations:** Division of Biomedical Statistics and Informatics, Department of Health Sciences Research, Mayo Clinic, 200 1st St SW, Rochester, MN 55905 USA; Epigenomics Translational Program, Center for Individualized Medicine, Mayo Clinic, Rochester, MN 55905 USA; Interdisciplinary Bioinformatics and Systems Biology Program, University of California at San Diego, La Jolla, CA 92093-0419 USA

**Keywords:** ChIP-Seq, Next-generation sequencing, Peak calling, Duplicate filtering, Irreproducible discovery rate

## Abstract

**Background:**

Chromatin immunoprecipitation (ChIP) followed by next-generation sequencing (ChIP-Seq) has been widely used to identify genomic loci of transcription factor (TF) binding and histone modifications. ChIP-Seq data analysis involves multiple steps from read mapping and peak calling to data integration and interpretation. It remains challenging and time-consuming to process large amounts of ChIP-Seq data derived from different antibodies or experimental designs using the same approach. To address this challenge, there is a need for a comprehensive analysis pipeline with flexible settings to accelerate the utilization of this powerful technology in epigenetics research.

**Results:**

We have developed a highly integrative pipeline, termed HiChIP for systematic analysis of ChIP-Seq data. HiChIP incorporates several open source software packages selected based on internal assessments and published comparisons. It also includes a set of tools developed in-house. This workflow enables the analysis of both paired-end and single-end ChIP-Seq reads, with or without replicates for the characterization and annotation of both punctate and diffuse binding sites. The main functionality of HiChIP includes: (a) read quality checking; (b) read mapping and filtering; (c) peak calling and peak consistency analysis; and (d) result visualization. In addition, this pipeline contains modules for generating binding profiles over selected genomic features, *de novo* motif finding from transcription factor (TF) binding sites and functional annotation of peak associated genes.

**Conclusions:**

HiChIP is a comprehensive analysis pipeline that can be configured to analyze ChIP-Seq data derived from varying antibodies and experiment designs. Using public ChIP-Seq data we demonstrate that HiChIP is a fast and reliable pipeline for processing large amounts of ChIP-Seq data.

**Electronic supplementary material:**

The online version of this article (doi:10.1186/1471-2105-15-280) contains supplementary material, which is available to authorized users.

## Background

Chromatin immunoprecipitation (ChIP) coupled with next-generation sequencing (ChIP-Seq) represents a powerful approach to identify genome-wide occupancy of transcription factors (TFs) and histone tail modifications [1]. The ENCODE and modENCODE consortia have generated an atlas of TF binding sites and histone modifications for 100+ cell types, including these from human and mouse [2].

ChIP-Seq data processing starts with the mapping of short reads to a genome reference. The mapped reads (alignments) are then used to generate signal tracks in a variety of formats (Wig, bigWig, bedGraph, or TDF) for data visualization. They are further used to identify regions showing significant enrichment over a control library like an IgG control generated using a non-specific IgG antibody, or an input control without using an antibody [2]. ChIP-Seq data shows three types of binding profiles: punctate binding, diffuse binding, and a mixture of both [3]. Sequence-dependent TFs and some histone modifications (such as H3K4me3) usually exhibit punctate binding sites of a few hundred base pairs in size. Comparatively, some other histone modifications display broad binding profiles that could spread over several hundred kilobases, such as H3K9me3, known to be associated with constitutive heterochromatin, and H3K36me3 associated with transcribed regions. The signals from RNA polymerase II peak at 5’ end of genes, and can extend over the body of transcribed genes, forming a mixture of sharp and diffuse binding profiles.

There are over thirty publicly available programs for peak calling [4]. Most of them focus on punctate binding profiles using either window scanning or aggregation of overlapping reads to identify peaks. A subset of these programs has been extensively evaluated on their sensitivity and specificity [4, 5]. Due to the variation of signal intensity, signal discontinuity within an entire binding domain and insufficient sequencing depth, it has been challenging to define the boundary of diffuse binding domains at high resolution [6]. Currently, only a few packages have been developed to analyze diffuse binding profiles [6–8]. Among them, SICER and RSEG are comparable for experiments with controls [8]. SICER is one of the best programs showing high accuracy in detecting broad binding regions from H3K36me3 [9].

The ENCODE consortium recommends that ChIP-Seq experiments have two biological replicates in order to assess data reliability. Based on the previous guideline, a ChIP-Seq experiment is considered to be reproducible if at least 75% of the peaks overlap between replicates; or top 40% of the peaks show >80% overlap [2]. A method called irreproducible discovery rate (IDR) has been developed, which measures the consistency between lists of ranked peaks from replicates [10]. It represents a more robust and consistent approach to identify highly reproducible peaks.

Several packages have been developed for downstream analysis of identified peaks. The most common analyses include the assignment of peaks to gene bodies or gene regulatory domains [11]; the generation of binding profiles over transcription start sites (TSSs) or other key genomic features [12–14]; the coverage of genomic features by peaks [12]; the testing of functional enrichment for peak-associated genes [13]; and motif finding [15]. Of these, a peak is usually assigned to a nearby gene based on a pre-defined cutoff for the maximal distance from peak center to gene start, which typically ranges from 2 to 50 kb but can be as far as 1 Mb [11]. This assignment introduces bias towards genes in closer vicinity of peaks and impacts subsequent tests for function enrichment.

A few pipelines have been developed to analyze ChIP-Seq data [13, 14, 16–18]. ChIPpeakAnno and seqMINER focus on the integration of ChIP-Seq data with genomic features [13, 14]. On the other hand, Fish the ChIPs (FC) [19] and a web server called Nebula [17] support read mapping; peak calling for punctate binding events; assignment of peaks to genes and data visualization. However, none of them provide functionality for the filtering of mapped reads; the identification of broad binding domains; the assessment of reproducibility; and the analysis of paired-end data.

To address this shortfall, the Highly Integrative Chromatin Immunoprecipitation (HiChIP) pipeline provides comprehensive analysis of ChIP-Seq data. HiChIP has the following features: (a) the analysis of both paired-end and single-end data; (b) filtering of mapped reads based on duplicate level, mapping quality score, genomic uniqueness, insertion size and orientation (for paired-end reads only); (c) the selection of an appropriate peak finder based on binding profile, with MACS [20] for punctate binding sites and SICER [6] for broad binding domains; (d) the implementation of the IDR package [10] to perform consistency analysis of punctate binding sites between replicates; and (e) downstream analysis, such as finding motif(s) from TF binding sites using MEME suite [15], generating binding profiles over key genomic features and calculating coverage of genomic features by peaks using CEAS [12], as well as assigning peaks to genes and testing for gene ontology (GO) enrichment using in-house tools. The integrative analysis allows bioinformaticians and investigators to spend less time on low-level data analysis and instead focus on data integration and interpretation.

## Implementation

**Figure 1:**
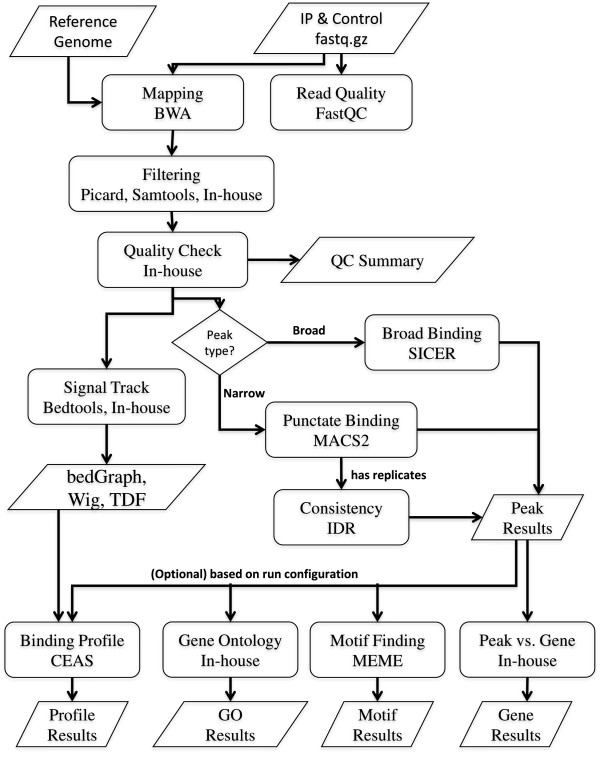
**Flowchart of HiChIP.** It contains five key functions (see implementation), developed by using both public tools and in-house scripts.

This ChIP-Seq analysis pipeline has been developed by integrating public packages with internally developed tools. It is intended for research purposes only. The core functions include: (a) read quality assessment and read mapping; (b) filtering of mapped reads and estimation of library complexity; (c) peak calling and identification of highly consistent peaks between replicates; (d) signal intensity estimation, normalization and visualization; and (e) annotation of peaks and binding profiles (Figure 1).

Since researchers may not always have immediate access to cluster resources, this pipeline allows either parallel processing of a large number of samples in a cluster or serial processing of multiple samples on a single machine. Detailed instructions about how to run HiChIP pipeline and how to use individual tools are described in the user manual available at: http://bioinformaticstools.mayo.edu/. Website containing license agreements for each of the public tools is also provided in the user manual.

### Test datasets

To test HiChIP performance, we used five public datasets in human, including single-end ChIP-Seq datasets targeting TFs NFKB and ER and histone mark H3K27me3; a paired-end ChIP-Seq dataset targeting TF RUNX1; and an ER chip-chip dataset. Each of the ChIP-Seq datasets includes both IP and control.

The NFKB datasets are from cell lines GM12878 and GM12891; each with two replicates for both IP and control. The FASTQ sequence files were downloaded from: http://hgdownload.cse.ucsc.edu/goldenPath/hg18/encodeDCC/wgEncodeYaleChIPseq.

The ER ChIP-Seq datasets include 18 libraries from five cell lines (MCF-7, ZR75-1, T-47D, BT-474, and TAM-R). Each cell line had 2–3 replicates for IP and a single control. We downloaded the BWA aligned BAM files from National Center for Biotechnology Information (NCBI) Gene Expression Omnibus (GEO) under accession GSE32222 [21].

The RUNX1 dataset is from an acute myeloid leukemia patient with the t(8;21) translocation [22]. The FASTQ files from one IP (GSM850826) and one control (GSM850828) were downloaded from NCBI GEO. Since the control library had only ~7.3 million pairs of reads, we downsized the total 18.8 million to 8 million pairs for the IP library.

The H3K27me3 datasets are from cell lines GM12878, HeLa S3 and MCF-7. GM12878 had two replicates in IP and one control library, while the other two cell lines each had two replicates for both IP and control. The FASTQ sequence files for GM12878 and HeLa S3 were downloaded from: http://hgdownload.cse.ucsc.edu/goldenPath/hg19/encodeDCC/wgEncodeBroadHistone and these for MCF-7 were from: http://hgdownload.cse.ucsc.edu/goldenPath/hg19/encodeDCC/wgEncodeSydhHistone.

The ER chip-chip data is generated from the MCF-7 cell line using the Affymetrix human tiling microarray. The dataset was downloaded from: http://research4.dfci.harvard.edu/brownlab//datasets/index.php?dir=ER_MCF7_whole_human_genome/.

### Read quality assessment

FastQC is a fast and flexible package for checking overall sequence quality (http://www.bioinformatics.babraham.ac.uk/projects/fastqc/). For each sample, FastQC reports the distribution of average per-base and per-read quality, as well as the level of duplication and possible sources of contaminations. If there is indication of abnormality in mapping results, such as low mapping rate, user can review read quality in the FastQC reports and try to improve the mapping rate by trimming low-quality bases or adaptor sequences in the reads.

### Read mapping algorithms

Several mapping software packages have been developed to map short reads to the reference genome [23]. BWA is a robust and fast short-read aligner, and has been widely used to map ChIP-Seq reads [24]. Novoalign (http://www.novocraft.com/main/index.php) is slower than BWA but is known to have higher sensitivity [23]. To decide which one to be implemented into the pipeline, we compared mapping rate between Novoalign and BWA on both single-end and paired-end ChIP-Seq data, and further assessed how the mapping difference might impact peak calling.

### Post-processing of mapped reads

After initial alignment, the mapped reads need to be further processed in order to improve peak calling sensitivity and specificity. The post-processing steps below address the issues of poorly mapped reads, duplicate reads and reads mapping to multiple locations.

### Reads with low mapping quality

It is a common practice to remove reads with low mapping quality. For single-end reads, HiChIP uses samtools [25] to filter out reads based on a user-defined mapping quality score threshold (default: 20). Mapped paired-end reads have three mapping states: both ends uniquely mapped; one of the ends uniquely mapped; both ends mapping to multiple locations (both have a zero mapping quality score). Samtools does not maintain the pairing information when performing mapping quality-based filtering for paired-end reads. Therefore, we provide a script to remove pairs of reads that have one or two ends below the mapping quality cutoff set by the user. The user can choose not to apply this filtering to the pairs of reads with the two ends mapping to multiple genomics locations (both have a quality score of “0” set by BWA). After the filtering, the proper pairing information will still be maintained.

### Duplicate reads

For ChIP experiments, the sequencing library is mostly generated from a much smaller amount of DNA compared to standard DNA or RNA sequencing. Duplicate reads that map to the same genomic location and strand are frequently present in ChIP-Seq datasets. For many applications, duplicate reads are removed as they are considered likely represent experimental artifacts. However, in the context of a ChIP-Seq experiment duplicate reads can also occur during the sequencing of identical DNA fragments in peak regions. In this case, duplicate reads contribute to peak identification and should not be removed.

Chen *et al*. reported that duplicate removal could improve the specificity of MACS peak calling [5]. Since the level of duplicate reads as artifacts versus as true signals cannot be well defined, Picard (http://picard.sourceforge.net/) is included in HiChIP to remove duplicate reads by default. A user can specify whether to remove duplicate reads. To reflect the level of duplicate reads, HiChIP uses a custom script to measure library complexity as the ratio between number of duplicate-filtered reads and the total number of uniquely mapped reads. As a guideline, library complexity needs to reach ~0.8 at a sequencing depth of 10 million mapped reads [2]. Low library complexity suggests suboptimal immunoprecipitation efficiency, a lack of sufficient starting material, PCR over-amplification, or a combination of these factors.

### Reads mapping to multiple genomic locations

In ChIP-Seq analysis, reads mapping to multiple genomic locations are often discarded [26]. Depending upon the nature of the studied epigenetic mark, this strategy may not be optimal in some cases. For instance, a substantial fraction of the H3K9me3 modification occurs in regions containing repetitive DNA sequences. In a survey of 12 H3K9me3 ChIP-Seq datasets from the ENCODE project (http://hgdownload.cse.ucsc.edu/goldenPath/hg19/encodeDCC/), between 16% and 28% of the mapped reads have multiple matches in the genome. It has also been shown that some TF binding sites are located in regions with poor mappability [26]. In such cases, excluding reads mapping to multiple genomic locations will decrease the sensitivity of peak detection in these less mappable regions.

HiChIP allows the user to specify whether to filter out reads matching multiple locations. For single-end reads, only uniquely mapped reads are kept by default, with the option to include one random match for reads mapping to multiple locations. For paired-end reads, we developed an in-house script to filter out undesired pairs. Only mapped pairs with appropriate insertion sizes and correct orientation are kept. Depending on the user’s specification, these reads are further processed to retain pairs belonging to one of the three types: (a) only uniquely mapped pairs; (b) pairs with at least one uniquely mapped end; or (c) pairs with at least one uniquely mapped end, plus a random match if both ends align to multiple locations. No currently available public tool provides equivalent flexibility in the filtering of mapped paired-end ChIP-Seq reads.

### Peak calling

There are two major ChIP-Seq binding profiles: punctate binding and diffuse binding. For punctate binding sites, peak calling identifies locations with maximum read density. For diffuse binding sites, the main goal is to define the boundary of individual binding domains. Therefore, different peak callers need to be used to take into account the differences in binding profile.

We used MACS to identify punctuate binding sites because of its high specificity and sensitivity [4, 5, 10]. MACS scans the genome for candidate regions and merges overlapping regions into peaks. It captures local signal fluctuation by modeling the background level as dynamic Poisson distribution.

The consistency of identified peaks can be assessed for punctate binding sites where replicates are available. In HiChIP, we implemented the IDR method to measure the consistency of peaks between replicates [10]. To prepare for IDR analysis, HiChIP combines mapped reads from IP replicates and those from control replicates into two merged datasets following the procedure proposed by Landt *et al*. [2]. The merged IP dataset is then split into two equally-sized pseudoreplicates after randomization; mapped reads from each IP are also split into two equally-sized pseudoreplicates. The true IP replicates, pseudoreplicates from each IP and merged IP dataset, merged IP and merged control datasets are then used in MACS peak calling. The consistency of resulting peaks is analyzed using the IDR procedure.

As suggested by Landt *et al*. [2], if a ChIP-Seq experiment has good reproducibility, the number of consistent peaks between true biological replicates and that between pseudoreplicates from merged IP should not differ by more than a factor of two. Similarly, the number of consistent peaks between pseudoreplicates from biological replicate 1 and that between pseudoreplicates from biological replicate 2 should also be within a factor of two. We added the IDR values (<1) estimated for shared peaks to the 4th column of the MACS output file with the ‘encodePeak’ extension. For replicate-specific peaks, an arbitrary value of ‘1’ is used instead. This will allow an easy extraction of consistent peaks at any user-specified IDR cutoff.

To identify diffuse binding sites, HiChIP leverages a widely used program called SICER [6]. SICER uses a clustering approach to define the boundary of diffuse binding sites. It identifies candidate sites of variable lengths based on a Poisson background model and links neighboring sites together if they are separated by gaps not exceeding a pre-defined gap size cutoff (gap size is 600 bp by default) and the whole domain is significantly enriched over the control [6]. SICER itself only provides filtering of binding regions based on FDR cutoff but not on fold change over the control. HiChIP further filters out candidate regions if the fold change is less than two.

### Putative cis-regulated genes

After peak calling, potential cis-regulated genes associated with peaks are identified, which is based on the maximum distance of peaks to the transcriptional start sites (TSSs) or translational end sites (TESs). By default, this distance is set at 10 kilobases.

### Data visualization

To enable visual inspection of discovered binding sites and their association with annotated genes or other genomic features, HiChIP generates files that can be visualized in a genome browser like the Integrative Genomics Viewer (IGV) [27]. CEAS needs a Wig file as an input. Since MACS version 2 does not generate a Wig file and SICER generates a Wig file with a relatively large span size (200 bp), we designed a module to generate bedGraph, Wig and tiled data format (TDF) files for data visualization.

To generate the bedGraph file, filtered reads in BAM format are first processed into bed format as follows. Single-end reads are extended by the average fragment length of the library (default 200 bp). For paired-end reads, the HiChIP pipeline keeps the first end and extends by the fragment length estimated from mapping positions of the two ends, rather than by the average fragment length of the library. Given the variability of fragment lengths across a complex genome like human genome, the use of actual coordinates of mapped pairs is expected to achieve better resolution in signal visualization. The bed file is then used to generate a bedGraph file by the genomeCoverageBed command from BEDTools [28].

The Wig file is generated from the bedGraph file, using an in-house script that computes the extended read coverage at a user-defined step size (default: 20 bp). The extended read coverage is normalized to a library size of one million mapped reads, and converted into the TDF format using the toTDF command from the igvtools package (http://www.broadinstitute.org/software/igv/igvtools). The normalized coverage in TDF format and identified peaks in bed format can be visualized by uploading files to IGV, or by opening the provided igv_session.xml file in IGV.

### Peak and binding profile annotation module

HiChIP includes three tools to annotate peaks and binding profiles. We use MEME [15] for identifying the TF binding motif; CEAS (Cis-regulatory Element Annotation System) [12] for generating binding profiles over key genomic features and for predicting possible genes regulated by cis-regulatory elements; and an in-house tool for calculating enrichment in gene ontology (GO) terms for peak-associated genes.

HiChIP selects top peaks as input for CEAS and MEME. Peaks used by CEAS are selected based on the pre-defined –log_10_ (*p* value) (for MACS peaks) or –log_10_ (FDR) cutoff (for SICER peaks). Since the detection of binding motif(s) using MEME is dependent upon the set of DNA sequences provided, attention needs to be paid to the cutoff for peak selection. By default the top 10% of peaks with the largest –log_10_ (*p* value) will be used. The HiChIP pipeline also allows the user to select a certain number of top peaks for motif discovery. CEAS uses normalized Wig files and peak files (bed format) as inputs, and performs binomial test for enrichment of binding over genomic regions such as gene promoters, gene bodies, exons and introns.

An in-house method is implemented to identify GO terms that are enriched in peak-associated genes. This method uses a similar approach as GREAT [11] that could not be integrated into our workflow, since the main functionality of GREAT is only available through web services. The lists of human and mouse genes with annotated GO terms were downloaded from the GREAT website (http://bejerano.stanford.edu/help/display/GREAT/Genes). For each gene annotated with at least one ontology term, the HiChIP pipeline first defines its regulatory domain as the region from upstream (U + UE) bp to downstream (D + DE) bp around the TSS, where the region from upstream U bp (default 5000) to downstream D bp (default 1000) represents the proximal regulatory domain, and UE and DE denote the maximum upstream and downstream extension, respectively. Binomial tests are then performed to identify a list of GO terms that are enriched in genes associated with peaks.

## Results and discussion

### Performance and output summary

We tested the pipeline performance on a Linux platform with an 8-core GenuineIntel CPU at 2.66 GHz. For a typical ChIP-Seq dataset containing a single IP and control library, each with 20–50 million pairs of reads, HiChIP takes 6–14 hours to complete at ~5-8 Gb memory usage.

The summary report provides links to the FastQC output files and an igv_session.xml file for data visualization. It also contains an html document that covers sample information, mapping summary, library complexity (Additional file 1: Table S1), peak summary, as well as histograms showing read pileup distribution within peaks. Depending on the user’s specification for peak calling, the pipeline will generate a list of peaks from MACS, MACS combined with IDR analysis, or SICER.

**Figure 2:**
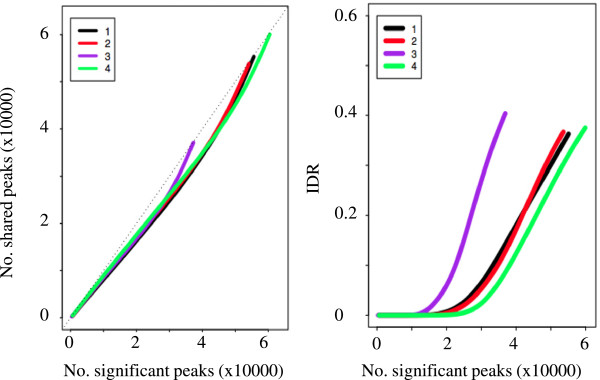
**Irreproducible discovery rate between replicates.** Left panel: number of significant peaks versus number of shared peaks called from both replicates. Right panel: number of significant peaks at different IDR cutoffs. 1: between biological replicates; 2: between pseudoreplicates from biological replicate 1, generated by randomly splitting the mapped reads into two equal-sized partitions; 3: between pseudoreplicates from biological replicate 2; 4: between pseudoreplicates from mapped reads merged from the two biological replicates. MACS called peaks at a *p* value cutoff of 1e-3, instead of the default 1e-5.

If IDR function is executed, the HiChIP pipeline will generate summary tables and a plot showing the number of reproducible peaks at different IDR cutoffs (Additional file 1: Table S2 and Additional file 1: Table S3; Figure 2). User can also get a conservative list and an optimal list of reproducible peaks at a given IDR cutoff (default 0.01). A conservative list represents the number of peaks shared between biological replicates, and an optimal list represents the number of shared peaks either between biological replicates or between pseudoreplicates from merged IP, whichever has more peaks.

To help with peak interpretation, the HiChIP pipeline generates a table that reports the closest genes (peak_vs_gene.xls). In addition, CEAS provides a report summarizing the percentage of peaks located in different regions such as promoters, gene upstream and downstream, UTRs, and provides plots showing binding profiles over selected genomic features (Additional file 2). MEME creates an html file that contains the most significant motif(s), and a text file with names of individual sequences from peak regions that contain a motif. Finally, the internally-developed GO enrichment test identifies the most significant terms enriched for peak-associated genes (Additional file 1: Table S4). We have included a word document to describe individual output files (HiCHIP_workflow_summary.doc).

### Comparison of BWA and Novoalign mapping

Novoalign represents one of the most accurate short-read aligners but is much slower than BWA [23]. Using two public ChIP-Seq datasets from TFs NFKB and RUNX1, we compared BWA with Novoalign on the mapping rate and its possible impact on MACS peak calling. For the 28-bp single-end NFKB dataset, BWA generated 3.2-4.8% more uniquely mapped reads than Novoalign for six of the eight libraries (Additional file 3). In two of the four IP libraries, more peaks containing the NFKB motif were identified from BWA-mapped reads (Table 1).

**Table 1:** **Number of NFKB peaks from BWA and Novoalign mapped reads**

Type	GM12878_NFKB_IP_rep1		GM12878_NFKB_IP_rep2		GM12891_NFKB_IP_rep1		GM12891_NFKB_IP_rep2	
	**A**	**B**	**A**	**B**	**A**	**B**	**A**	**B**
Overlapped peaks	1512	1513	9942	9960	2257	2264	6082	6082
Unique peaks w/ motif	176	18	409	90	88	19	36	30
Unique peaks w/o motif	134	28	590	249	442	74	21	7
Total peaks	1822	1559	10941	10299	2787	2357	6139	6119

Peaks were identified using MACS (−f BAM -g hs --keep-dup 1 -q 0.01) and motif finding by MEME suite (−dna -mod zoops -nmotifs 5 -minw 10 -maxw 20 -maxsize 999999999 -revcomp). Shown were the number of overlapped peaks between BWA mapped reads (A) and Novoalign mapped reads (B), and number of aligner-specific peaks with or without NFKB binding motif. ChIP-Seq from each cell line had two biological replicates (rep1 and rep2).

For the 45-bp paired-end RUNX1 dataset, Novoalign mapped about 7% more reads uniquely to the genome than BWA (Table 2). This resulted in the identification of 224 unique peaks only from Novoalign-mapped reads, versus 103 unique peaks only from BWA-mapped reads (Table 3). However, the number of unique peaks with the RUNX1 binding motif was almost identical: 86 peaks for Novoalign-mapped read and 85 for BWA-mapped reads. Since BWA performs better than Novoalign on mapping single-end reads, similarly on mapping paired-end reads and BWA is >10x faster, HiChIP uses BWA as the aligner.

**Table 2:** **BWA and Novoalign mapping of paired-end reads**

Library	Type	Total (million)	BWA			Novoalign		
			**A**	**B**	**C**	**A**	**B**	**C**
GSM850526	IP	8	86	5.09	8.91	92.71	4.75	2.54
GSM850528	Control	7.29	82.32	7.09	10.59	89.68	6.62	3.7

The 45-bp paired-end ChIP-Seq data were from TF RUNX1. Raw reads were mapped to hg19 by both BWA and Novoalign. BWA parameters are: bwa aln -o 1 -l 32 -t 4 -k 2 and bwa sampe -n 10 -a 500 -o 10000 -N 10 -s. Novoalign parameters are: novoalign -i PE 250,30 -r Random --hdrhd off -c 1 -d reference.nix -F STDFQ -f end1.fastq end2.fastq -o SAM, where reference.nix is the reference sequence index file. A: percentage of pairs with at least one uniquely mapped end; B: percentage of pairs with multiple mapping locations; C: others, including unmapped pairs, pairs with only one mapped end and improperly mapped pairs with small insertion size or wrong orientation.

**Table 3:** **RUNX1 peaks from BWA and Novoalign mapped reads**

Type	BWA	Novoalign
Overlapped peaks	818	809
Unique peaks w/ motif	85	86
Unique peaks w/o motif	28	138
Total peaks	931	1033

Peaks were called by MACS (−f BAM -g hs --keep-dup all -q 0.01), after removal of duplicates by Picard. Motif was identified by MEME suite.

### Comparison of ChIP-Seq and chip-chip peaks

To check how many ChIP-Seq peaks can be found in chip-chip experiments, we compared ER peaks from three ChIP-Seq libraries in MCF-7 [21] to a list of 4,621 chip-chip peaks generated from MCF-7 using the Affymetrix human tiling microarrays [29]. Of the 4,621 chip-chip peaks, 4,373 (94.6%) occurred in all three ChIP-Seq replicates and 132 (2.9%) occurred in only one or two replicates. This left 117 chip-chip peaks not overlapped by any ChIP-Seq peaks (Table 4). To examine whether the 117 peaks are chip-chip specific, or ChIP-Seq peaks but simply missed by the selected three ChIP-Seq libraries, we compiled a total of 120,158 ER peaks from an additional 10 ChIP-Seq libraries [21]. Only 15 of the 117 chip-chip peaks showed overlap with peaks from these 10 libraries, suggesting that the majority of the 117 peaks are chip-chip specific or false positives.

**Table 4:** **ER ChIP-Seq and chip-chip peaks in MCF-7 cell line**

ChIP-Seq		Chip-chip			
Library	Total peaks	Total peaks	Overlapped peaks	Unique peaks	Overlap (%)
IP_1	69390	4621	4492	129	97.2
IP_2	46688	4621	4391	230	95.0
IP_3	57657	4621	4450	171	96.3

A list of 4,621 highly confident (*p* value < =1e-5) chip-chip peaks was extracted from the file “ER_binding_p-value.xls” downloaded from http://research4.dfci.harvard.edu/brownlab//datasets/index.php?dir=ER_MCF7_whole_human_genome/. ChIP-Seq peaks were identified by MACS at the *q* value cutoff of 0.01. Both ChIP-Seq and chip-chip analysis used hg18 reference sequence.

### The impact of duplicate removal on TF peak calling

To assess how duplicate removal might impact peak calling, we compared ChIP-Seq peaks identified in the three ER libraries before and after duplicate removal [21]. Between 238 and 1,689 peaks were identified only from reads before duplicate removal, versus 3,348 to 7,206 peaks only from reads after duplicate removal (Table 5). We hypothesized that duplicate reads add noise to the data and duplicate filtered reads are more likely associated with ER binding sites. To validate this hypothesis, we first used MEME to identify ER binding motif from the 100-bp sequences centered on peaks. Between 1,121 and 2,552 (26–35.4%) of the unique peaks identified from duplicate-filtered reads contained ER binding motif, versus 35–670 unique peaks from reads without duplicate removal (Table 5). Also, 57.5-81.8% of the unique peaks called after duplicate removal were shared by the 120,158 ER peaks from the other 10 libraries [21]. These findings indicate that duplicate removal improves the sensitivity of MACS peak calling for ER ChIP-Seq data and is a critical step in the HiChIP pipeline [5].

**Table 5:** **Number of ER peaks from MCF-7 cell line**

Type	IP_1 vs. control		IP_2 vs. control		IP_3 vs. control	
	A	B	A	B	A	B
Unique reads (million)	16.9	22.27	21.08	22.66	25.36	29.15
Overlapped peaks	62184	62407	41455	41551	54309	54354
Unique peaks	7206	1689	5233	238	3348	857
Unique peaks w/ motif	2552	670	1359	35	1121	362
Unique peaks w/o motif	4654	1019	3874	203	2227	495
Total peaks	69390	64096	46688	41789	57657	55211

Peaks were called by MACS **(−**f BAM -g hs --keep-dup all -q 0.01**)** from BWA-mapped reads (to hg18). Motif was identified using MEME as described above. A: number of ER peaks identified by this pipeline, using duplicate-filtered reads (by Picard); B: number of peaks from reads without duplicate removal. IP_1 to IP_3 are three replicates, IP_1: GSM798423, IP_2: GSM798424, IP_3: GSM798425; control (input): GSM798440.

**Figure 3:**
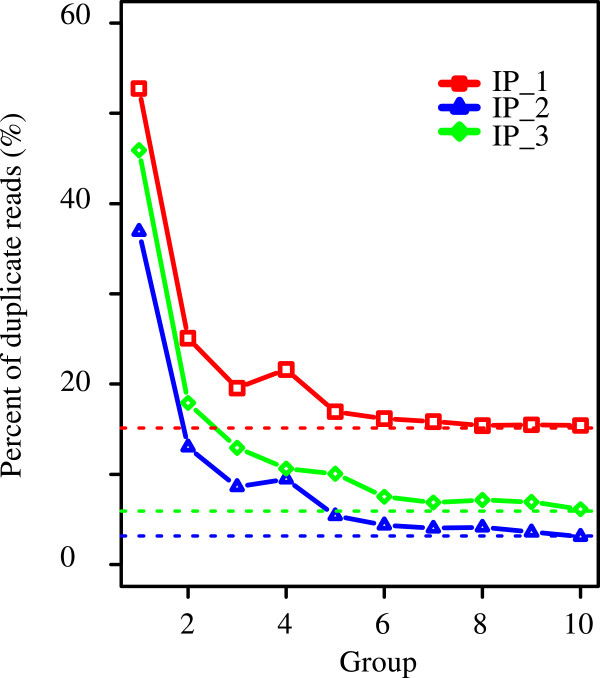
**Peak** ***p*** **value versus level of duplicate reads in three ER ChIP-Seq libraries.** Peaks were ranked based on –log_10_ (*p* value) in descending order and split into 10 groups (*x*-axis) of equal size. The y-axis represents the level of duplicate reads. The horizontal dashed lines indicate the level of duplication from non-peak regions, which are regions not covered by peaks +/−100 bp.

The two approaches differed only in the filtering of duplicates, suggesting that the identification of unique peaks in the three libraries is due to this filtering. To support this inference, we first checked the level of duplication in the three libraries. Indeed, library IP_1 (GSM798423) that had the most extra peaks (7,206, Table 5) after duplicate removal contained up to 24% duplicates, versus only 7% in IP_2 (GSM798424) and 13% in IP_3 (GSM798425, based on Table 5). To further illustrate the distribution of duplicates from peak versus that from non-peak regions, we extended each peak by 100 bp at both sides and defined the remaining regions as non-peak regions. These non-peak regions showed the basal level of duplication, which was 14.5% in IP_1, and <6% in the other two libraries (Table 6; Figure 3). Duplicates were heavily biased toward peak regions, as compared to non-peak regions. In all three libraries, peak regions together covered <1% of the mappable genome but harbored >60% of the duplicates (Table 6).

**Table 6:** **Duplicate level in three ER ChIP-Seq libraries**

Library	Type*	Size (Mb)	Non-duplicates (M)	Duplicates (M)	Sum (M)	Duplicates (%)	Portion (%)**
IP_1	All peaks	17.08	4.34	3.24	7.58	42.73	60.23
IP_1	Top peaks	3.33	1.92	2.54	4.46	57.03	47.3
IP_1	Non-peak	3049.89	12.27	2.08	14.35	14.51	38.71
IP_1	Total	3080.44	16.9	5.38	22.27	24.13	100
IP_2	All peaks	12.54	2.94	1.1	4.04	27.3	69.79
IP_2	Top peaks	2.31	1.2	0.9	2.11	42.84	57.12
IP_2	Non-peak	3058.77	17.9	0.47	18.36	2.54	29.55
IP_2	Total	3080.44	21.08	1.58	22.66	6.98	100
IP_3	All peaks	15.94	4.87	2.69	7.56	35.53	70.87
IP_3	Top peaks	3.02	1.97	2.05	4.02	51.04	54.1
IP_3	Non-peak	3053.24	20.11	1.08	21.19	5.09	28.46
IP_3	Total	3080.44	25.36	3.79	29.15	13.01	100

*The non-peak regions are these after excluding regions from −100 bp to +100 bp of all peaks; the top peaks refer to the top 10% of the peaks with the smallest *p* values.

**Percentage over total duplicates in the library.

To test whether the abundance of duplicates is correlated with the confidence level of peaks, for each library we split the *p*-value-sorted peaks into 10 equal-sized groups, with peaks in the first group having the lowest *p* values. The top 10% of the peaks (in the first group) had a duplication rate between 42.8% to 57%, containing roughly half of the total duplicates (Table 6; Figure 3). In contrast, the bottom 70% of the peaks (groups 4 to 10) had much reduced duplication rate (Figure 3). Our analysis suggested that, while filtering of duplicates contributes to the identification of extra peaks, it reduces the signal intensity to a much greater extent for the most significant peaks. The latter will impact the test for differential binding between different IPs.

We further used the TF RUNX1 dataset to investigate how duplicate removal might impact peak calling from paired-end data. The RUNX1 IP had up to 67% duplicates identified by the Picard MarkDuplicates command. We used MACS to call 931 peaks (Table 3) from duplicate-filtered reads and 16 times more peaks (14,916) from reads prior to duplicate removal. Of the 14,013 peaks not overlapping the 931 peaks, only 73 (0.5%) contained the RUNX1 binding motif. This suggests that the vast majority of the 14,013 unique peaks from reads without duplicate removal represent false positives. This result, together with the ER ChIP-Seq results, supports duplicate removal when analyzing TF ChIP-Seq data.

### The impact of duplicate removal on H3K27me3 peak calling

**Figure 4:**
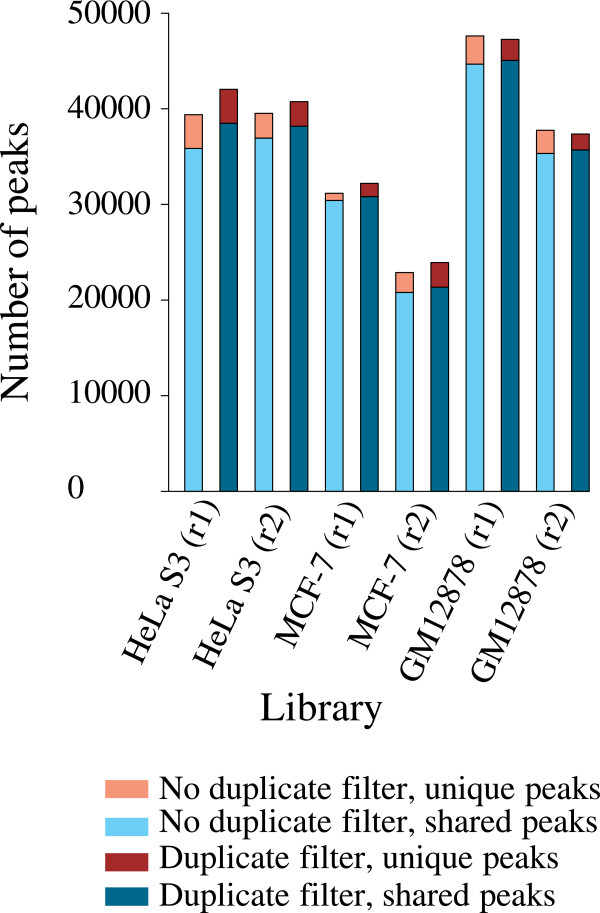
**Summary of peak calling for H3K27me3 ChIP-Seq.** H3K27me3 ChIP-Seq data were downloaded from the ENCODE project (see implementation) and duplicates were filtered out by Picard. Each cell line has two biological replicates (r1 and r2). SICER software was used to call peaks using both duplicate-filtered reads and reads without duplicate removal.

**Figure 5:**
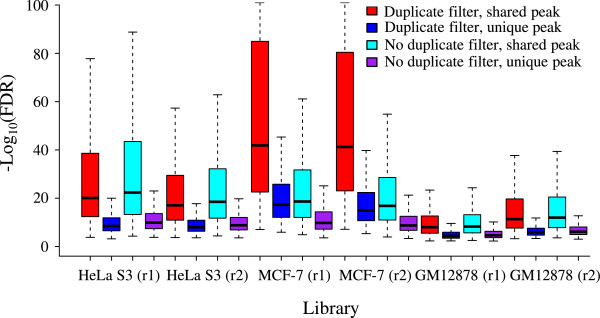
**Box plot of FDR from shared and unique H3K27me3 peaks.** Peaks were called from duplicate-filtered reads and also from reads without duplicate filtering. Peaks shared between the two methods were randomly sampled to generate the same number of peaks as the unique peaks and used in the plot. The *y*-axis represents –log_10_ (FDR). The two biological replicates are indicated by r1 and r2, respectively.

We next explored whether duplicate removal improves peak calling for ChIP-Seq data showing broad binding profile. We analyzed H3K27me3 histone modification dataset. H3K27me3 is catalyzed by the polycomb repressive complex 2 (PRC2) and largely associated with transcription repression. FASTQ files for six single-end H3K27me3 ChIP libraries and five control libraries were downloaded from the ENCODE project, mapped by BWA, and duplicates filtered by Picard (Additional file 1: Table S5, also see Duplicate reads). These libraries include 11.2-34.6 million uniquely mapped reads with 2-8% duplicates. We identified 23,918-47,260 binding sites from duplicate-filtered reads and 22,869-47,611 binding sites from reads without duplicate filtering (Figure 4). For each library, the lists of peaks with and without duplicate removal overlapped by 89-98%, with roughly the same number of peaks unique to each list. We observed that the unique peaks had much higher FDR than the peaks shared by the two lists (Figure 5), suggesting that these unique peaks represent less confident sites with low H3K27me3 occupancies.

**Figure 6:**
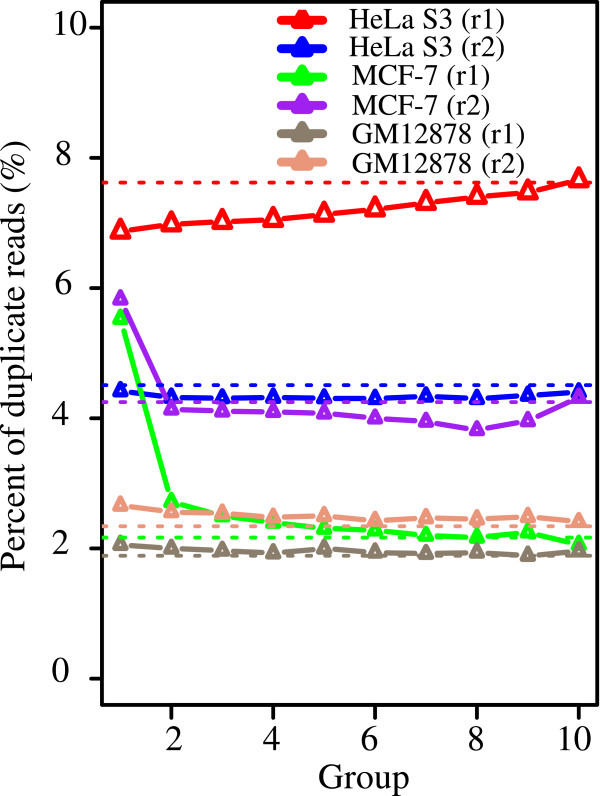
**Peak FDR versus level of duplicate reads in six H3K27me3 ChIP-Seq libraries.** Peaks were ranked based on –log_10_ (FDR) in descending order and split into 10 groups (*x-*axis) of equal size. The *y*-axis represents the level of duplicate reads. The horizontal dashed lines indicate the level of duplication from non-peak regions, as defined in Figure 3. The two biological replicates are indicated by r1 and r2, respectively.

To investigate whether duplicates are enriched in highly confident peaks as previously seen in the ER ChIP-Seq dataset (Figure 3), we split peaks into 10 groups based on ascending order of FDR and checked the level of duplicates per group (Figure 6). Strikingly, the H3K27me3 ChIP-Seq data had comparable levels of duplicates between peak and non-peak regions; the top peaks generally did not show enrichment of duplicates (Figure 6). Unlike in the TF ChIP-Seq data showing narrow binding sites, duplicates are not particularly enriched in broad peaks called in the H3K27me3 dataset. These results indicate that duplicate removal appears to have less impact on peak calling for diffuse binding profile.

### IDR analysis

We tested IDR analysis using three ER ChIP-Seq libraries, which include two biological replicates for IP (IP_1 and IP_2) and a single control [21] (Tables 4 and 5). To call both significant and insignificant peaks in order to identify an appropriate IDR cutoff, we used a less stringent *p* value cutoff (1e-3) in MACS peak calling. When plotting the number of reproducible peaks over different IDR values, a clear transition was observed from highly reproducible peaks to poorly reproducible peaks (Figure 2). At the IDR cutoff of 0.01 (default), there were 22,382 and 14,286 consistent peaks between the two pseudoreplicates of IP_1 and IP_2, respectively, with a ratio of 1.6 (22382/14286). We identified 26,971 consistent peaks between two pseudoreplicates from merged IP and 21,224 consistent peaks between replicates IP_1 and IP_2, with a ratio of 1.3. In both cases, the ratio is less than 2, indicating good reproducibility between IP_1 and IP_2 (Figure 2; Additional file 1: Table S3). If two replicates show poor reproducibility (ratio >2), then it is necessary to generate a third replicate to validate the reliability of identified peaks.

## Conclusions

HiChIP is a comprehensive ChIP-Seq data analysis pipeline with more than 10 functions (Figure 1). It performs read mapping, peak calling for punctate and diffuse binding sites and downstream functional analysis. To enhance the quality of peak calling, HiChIP includes options for filtering out less reliably mapped reads to reduce noise. It also includes IDR analysis to identify a list of reproducible peaks between replicates. It provides a consistent and configurable method to assist the user to run this pipeline.

By applying HiChIP to publicly available single-end ER ChIP-Seq datasets we found that filtering of duplicates increases the sensitivity of MACS peak calling but heavily underestimates enrichment levels for the most significant peaks. For the paired-end RUNX1 ChIP-Seq data, the vast majority of the peaks called only from reads without duplicate removal represent false positives. These results suggest the necessity of enabling duplicate filtering for TF peak calling and using all mapped reads for estimating enrichment level and identifying differential binding sites. In contrast, duplicate filtering has less impact on peak calling from marks showing broad binding profile like H3K27me3.

Although HiChIP has combined several methods to enhance the preprocessing and annotation of ChIP-Seq data, several challenges remain that need to be addressed in the future. For example, it is still difficult to define the boundary of diffuse binding sites at high resolution and to identify the direct target genes of TF binding sites and histone modifications. As new or improved methods become available, the modular design of HiChIP will enable their smooth integration into the existing pipeline.

## Availability and requirements

**Project name:** HiChIP: A high-throughput pipeline for integrative analysis of ChIP-Seq data

**Project home page:**http://bioinformaticstools.mayo.edu/

**Operating system:** 64-bit Linux (The program has been tested on Centos)

**Programming language:** Shell, Perl and R

**Other requirements:**

- JAVA version 1.6.0_17 or higher
- Perl version 5.10.0 or higher
- Python version 2.7 or higher
- Python-dev
- Cython and Numpy python modules
- R version 2.14.0 or higher
- FastQC version 0.10 or higher
- BWA version 0.5.9 or higher
- MACS version 2.0.10 or higher
- SICER version 1.1
- IGVTools version 2.3.16
- Samtools version 0.1.19
- MEME version 4.8.1
- CEAS version 1.0.2
- Picard version 1.97
- BEDTools version 2.17.0

## Electronic supplementary material

Additional file 1: Table S1: Complexity of NFKB ChIP-Seq IP and control libraries. **Table S2.** Number of consistent peaks at different IDR values. ER ChIP-Seq datasets from MCF-7 cell line were used. **Table S3.** Summary of IDR analysis of ER ChIP-Seq data. **Table S4.** A snapshot of output from GO enrichment analysis. ER ChIP-Seq library IP_1 (GSM798423, MCF-7 cell line) was used. **Table S5.** Duplicate level in six H3K27me3 ChIP-Seq libraries. (XLS 68 KB)

Additional file 2: **Snapshot of output from CEAS analysis.** Reads mapping to chromosome 1 from libraries IP_1 and input were used [21]. Top panel: the distribution of peaks in 11 genomic features; bottom panel: average binding profiles around TSS +/−2 kb for all the RefGene and for two user-provided gene lists. (PPT 569 KB)

Additional file 3: **BWA versus Novoalign in mapping single-end reads.** The 28-bp ChIP-Seq reads from eight libraries of TF NFKB were downloaded from UCSC (http://hgdownload.cse.ucsc.edu/goldenPath/hg18/encodeDCC/wgEncodeYaleChIPseq/). Reads were mapped to the human genome reference hg19 using BWA and Novoalign. BWA parameters are: bwa aln -o 1 -l 32 -t 4 -k 2 and bwa samse -n 10 -f; novoalign parameters are: Novoalign -r Random --hdrhd off -c 1 -d reference.nix -F STDFQ -f end1.fastq -o SAM. BWA mapping: libraries 1, 3, 5, 7, 9, 11, 13, and 15; Novoalign mapping: libraries 2, 4, 6, 8, 10, 12, 14, and 16. Numbers in parentheses represented number (in million) of total raw reads and uniquely mapped reads, respectively. For six of the eight libraries, BWA increased uniquely mapped reads by 3.2-4.8%. 1: GM12878_Input_IgG_rep1 (27.42, 17.29) 2: GM12878_Input_IgG_rep1 (27.42, 16.24). 3: GM12878_Input_IgG_rep2 (18.33, 10.96) 4: GM12878_Input_IgG_rep2 (18.33, 10.23). 5: GM12878_NFKB_IP_rep1 (25.17, 18.93) 6: GM12878_NFKB_IP_rep1 (25.17, 18.13). 7: GM12878_NFKB_IP_rep2 (17.2, 13.24) 8: GM12878_NFKB_IP_rep2 (17.2, 12.63). 9: GM12891_Input_IgG_rep1 (17.05, 8.45) 10: GM12891_Input_IgG_rep1 (17.05, 7.63). 11: GM12891_Input_IgG_rep2 (12.35, 6.24) 12: GM12891_Input_IgG_rep2 (12.35, 6.26). 13: GM12891_NFKB_IP_rep1 (29.25, 13.9) 14: GM12891_NFKB_IP_rep1 (29.25, 12.66). 15: GM12891_NFKB_IP_rep2 (30.63, 16.05) 16: GM12891_NFKB_IP_rep2 (30.63, 16.09). (PPT 152 KB)
