# Supplementary figures and images for: HiChIP: a high-throughput pipeline for integrative analysis of ChIP-Seq data
Source: BMC Bioinformatics. 2014 Aug 15;15(1):280. doi: 10.1186/1471-2105-15-280 (PMC4152589; doi:10.1186/1471-2105-15-280)

## Slide 1
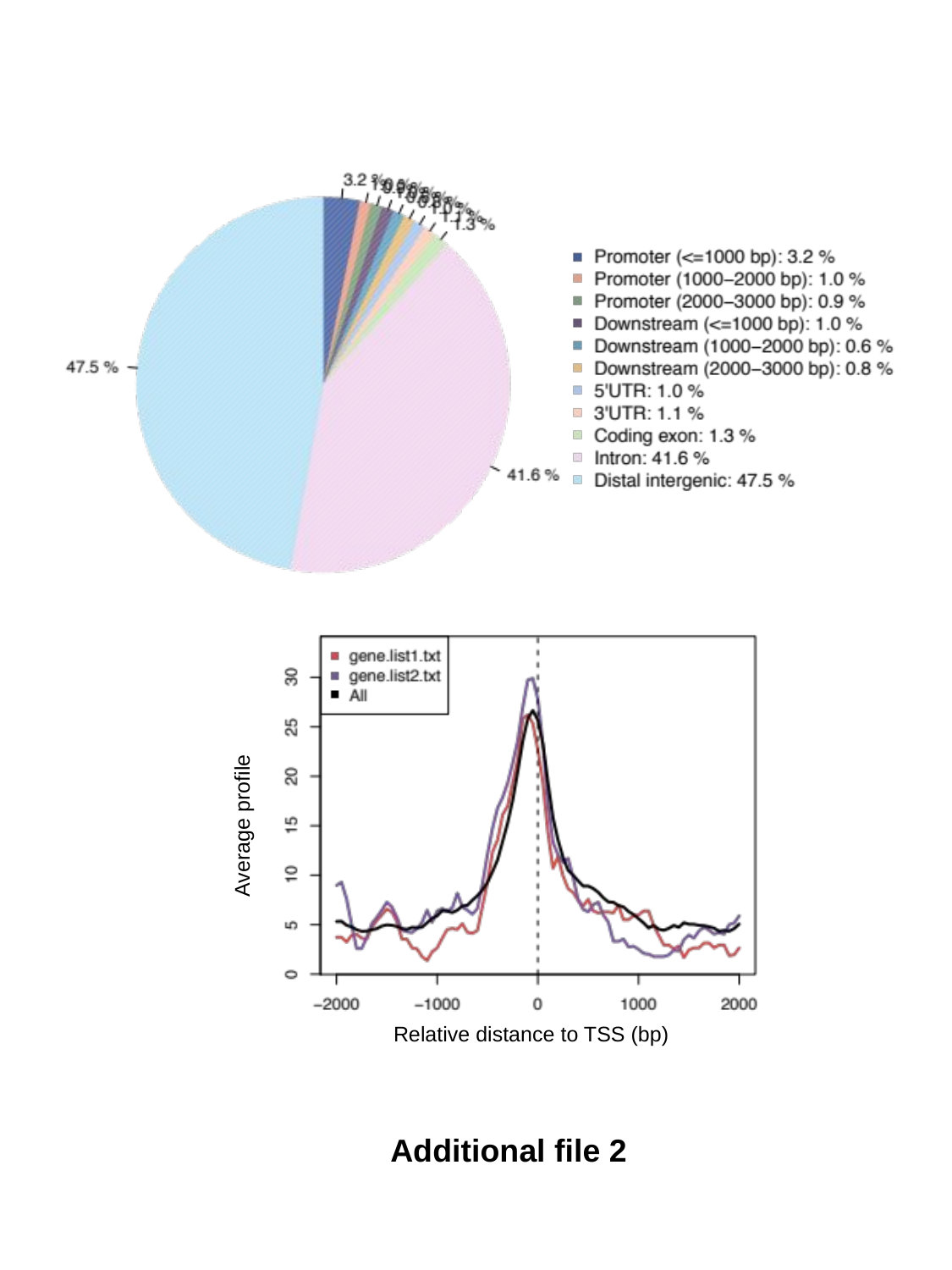

Average profile
Relative distance to TSS (bp)
Additional file 2

Supplement: Supplementary file 2 — Additional file 2: Snapshot of output from CEAS analysis. Reads mapping to chromosome 1 from libraries IP_1 and input were used [21]. Top panel: the distribution of peaks in 11 genomic features; bottom panel: average binding profiles around TSS +/−2 kb for all the RefGene and for two user-provided gene lists. (PPT 569 KB) [file 12859_2014_6551_MOESM2_ESM.ppt]

## Slide 1
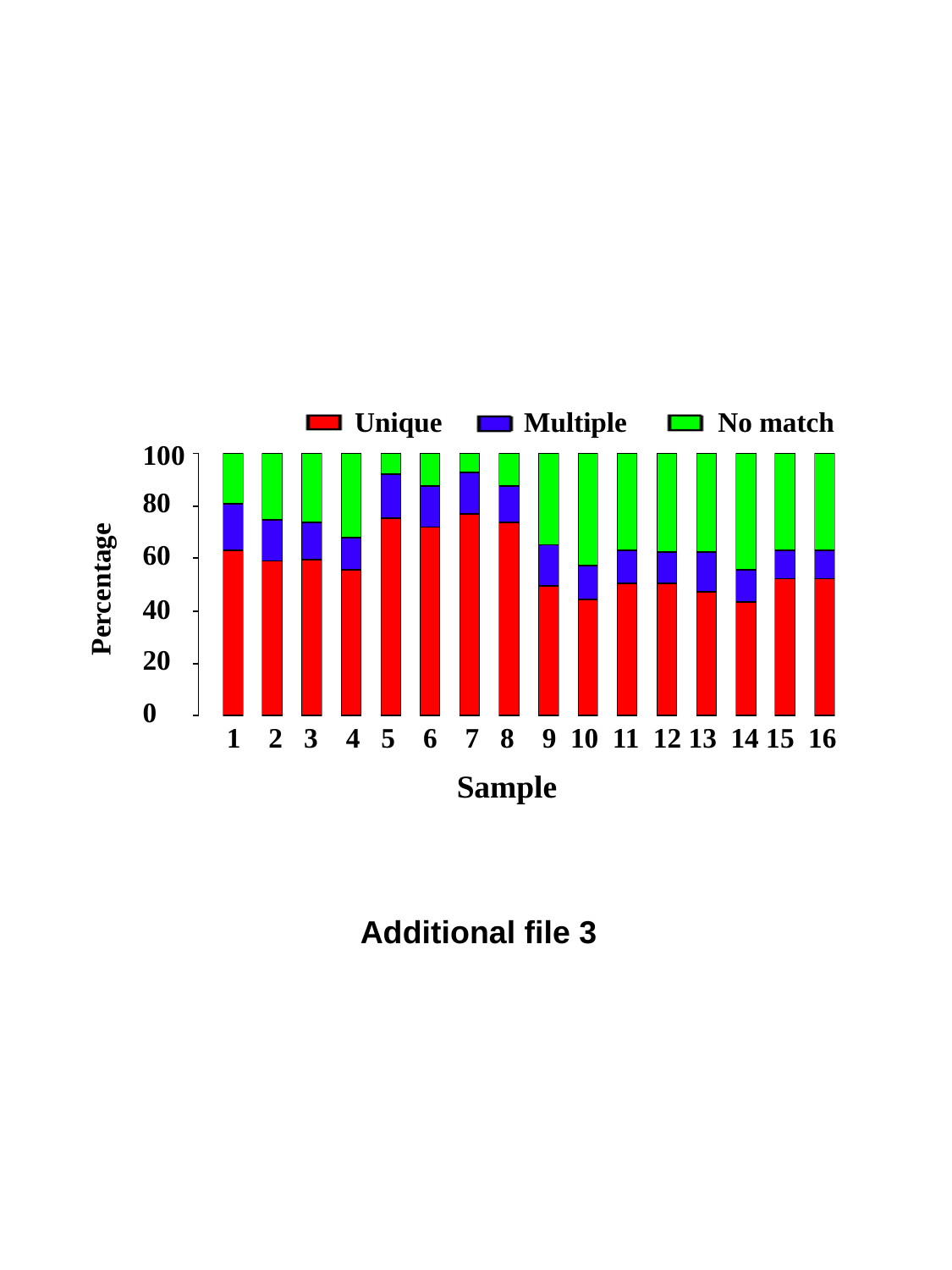

Unique
Multiple
No match
100
80
60
Percentage
40
20
0
1 2 3 4 5 6 7 8 9 10 11 12 13 14 15 16
Sample
Additional file 3

Supplement: Supplementary file 3 — Additional file 3: BWA versus Novoalign in mapping single-end reads. The 28-bp ChIP-Seq reads from eight libraries of TF NFKB were downloaded from UCSC (http://hgdownload.cse.ucsc.edu/goldenPath/hg18/encodeDCC/wgEncodeYaleChIPseq/). Reads were mapped to the human genome reference hg19 using BWA and Novoalign. BWA parameters are: bwa aln -o 1 -l 32 -t 4 -k 2 and bwa samse -n 10 -f; novoalign parameters are: Novoalign -r Random --hdrhd off -c 1 -d reference.nix -F STDFQ -f end1.fastq -o SAM. BWA mapping: libraries 1, 3, 5, 7, 9, 11, 13, and 15; Novoalign mapping: libraries 2, 4, 6, 8, 10, 12, 14, and 16. Numbers in parentheses represented number (in million) of total raw reads and uniquely mapped reads, respectively. For six of the eight libraries, BWA increased uniquely mapped reads by 3.2-4.8%. 1: GM12878_Input_IgG_rep1 (27.42, 17.29) 2: GM12878_Input_IgG_rep1 (27.42, 16.24). 3: GM12878_Input_IgG_rep2 (18.33, 10.96) 4: GM12878_Input_IgG_rep2 (18.33, 10.23). 5: GM12878_NFKB_IP_rep1 (25.17, 18.93) 6: GM12878_NFKB_IP_rep1 (25.17, 18.13). 7: GM12878_NFKB_IP_rep2 (17.2, 13.24) 8: GM12878_NFKB_IP_rep2 (17.2, 12.63). 9: GM12891_Input_IgG_rep1 (17.05, 8.45) 10: GM12891_Input_IgG_rep1 (17.05, 7.63). 11: GM12891_Input_IgG_rep2 (12.35, 6.24) 12: GM12891_Input_IgG_rep2 (12.35, 6.26). 13: GM12891_NFKB_IP_rep1 (29.25, 13.9) 14: GM12891_NFKB_IP_rep1 (29.25, 12.66). 15: GM12891_NFKB_IP_rep2 (30.63, 16.05) 16: GM12891_NFKB_IP_rep2 (30.63, 16.09). (PPT 152 KB) [file 12859_2014_6551_MOESM3_ESM.ppt]
